# Factors associated with social inclusion of parents of children with autism spectrum disorder: a multicenter cross-sectional study in China

**DOI:** 10.1186/s13034-026-01065-w

**Published:** 2026-03-14

**Authors:** Binbin Ji, Bohan Zhang, Siyu Chen, Yun Chen, Wanrui Wei, Longgang Zhao, Jing Li, Zhao Ni, Xiaojian Jiang

**Affiliations:** 1 School of Nursing, Hunan University of Chinese Medicine, Changsha, 410208 Hunan China; 2 School of Nursing, Beijing University of Chinese Medicine, Beijing, 100102 China; 3 Centre for Health Behaviours Research, Jockey Club School of Public Health and Primary Care, Faculty of Medicine, The Chinese University of Hong Kong, Hong Kong, China; 4 School of Nursing, Yale University, New Haven, CT 06477 USA; 5 Xiangya School of Nursing, Central South University, Changsha, 410008 Hunan China

**Keywords:** Autism spectrum disorder, Social inclusion, Parents, Children

## Abstract

**Background:**

Social inclusion of parents of children with autism spectrum disorder (ASD) affects their well-being and caregiving capacity. However, evidence on its factors remains limited in China. This study aimed to assess the social inclusion of parents of children with ASD and identify key factors, with attention to gender differences.

**Methods:**

1007 parents were enrolled from 33 rehabilitation centers in Hunan, China. Self-reported psychometric scales and a revised Chinese Social Inclusion Scale were used. Guided by the ecological model, univariate and multivariate regression analyses were performed to identify factors associated with social inclusion. Subgroup analyses and interaction effect tests were carried out to explore potential gender differences.

**Results:**

At the individual level, higher education (B = 1.764, *P* = 0.02), self-esteem (B = 0.472, *P* < 0.001), and hope (B = 0.139, *P* = 0.03) demonstrated positive associations with social inclusion. At the external level, intergroup relations (B = 0.271, *P* = 0.001) and social support (B = 0.238, *P* < 0.001) showed significant positive associations, while perceived discrimination (B=-0.189, *P* < 0.001) exhibited a significant negative association. Gender subgroup analyses showed fathers’ inclusion was mainly influenced by self-esteem and social support, while mothers’ patterns aligned with the overall group, except hope was not significant.

**Conclusions:**

Both psychological and social factors influence the social inclusion of parents of children with ASD, with gender-specific patterns. To enhance social inclusion of parents, healthcare providers could consider gender-responsive approaches that strengthen psychological resources while addressing external barriers.

**Supplementary Information:**

The online version contains supplementary material available at 10.1186/s13034-026-01065-w.

## Background

Autism spectrum disorder (ASD) is an early-onset, often lifelong neurodevelopmental condition characterized by persistent deficits in social-communication skills and restricted, repetitive behaviors [1]. In China, approximately 0.7% of children under the age of 14 are diagnosed with ASD, totaling over two million cases, making it the most prevalent neurological and developmental disorder among children [2]. Although public awareness of ASD has increased, societal understanding of its impact on families remains limited. Parents of children with ASD often face unique social challenges that go beyond caregiving responsibilities.

Social inclusion is a multifaceted and dynamic process that involves equal opportunities for social participation, both in terms of objective engagement and subjective experience [3]. Understanding the factors that influence parents’ social inclusion is critical because their well-being directly affects their ability to raise their children and to address the challenges associated with ASD. Numerous studies have highlighted the social inclusion challenges faced by families and their children with ASD [4–6]. Parents, especially mothers, often make significant personal sacrifices, such as quitting their jobs or reducing social activities, to provide full-time care [7]. These constraints, in turn, limit their own opportunities for community involvement and social inclusion. Additionally, when parents feel societal prejudice, discrimination, or a lack of understanding, they may choose to withdraw from social activities to shield their children from external pressure and exclusion [8].

Social inclusion is essential for parents’ well-being and mental health, as it fosters a sense of belonging and alleviates feelings of isolation, ultimately enhancing their overall quality of life [9–11]. Moreover, parents’ social inclusion plays a crucial role in shaping their children’ s social inclusion [12]. Socially engaged parents are more likely to access resources, receive community support, and engage in advocacy opportunities, all of which can facilitate their children’s participation in social activities, and improve their children’s outcome [13]. The reciprocal nature of social inclusion within families is particularly pronounced in the Chinese cultural context, where strong family interdependence can exacerbate these effects [14]. Therefore, addressing the social inclusion of parents of children with ASD in China is of great significance.

While extensive research has examined the social inclusion challenges of children with ASD, studies focusing on their parents remained limited [15–17]. In interventions aimed at improving the social inclusion of children with ASD, such as social skills training, parents often served as implementers [18]. However, their own social inclusion is frequently overlooked. This gap in the literature underscores the need to examine the current state of parents’ social inclusion and its associated factors, as these experiences not only directly impact parents’ well-being [9–11] but also indirectly shape their children’s social inclusion [12] and holistic development, including emotional, social, and cognitive growth [13].

Existing research identifies multiple factors influencing social inclusion, including personal characteristics, psychological attributes (e.g., self-esteem and hope) [19, 20], parenting-related factors (e.g., sense of competence) [21], and broader social factors (e.g., intergroup relations, family function, social support, and perceived discrimination) [22–25]. However, few studies have focused on parents of children with ASD, and many have examined only a limited range of factors, lacking a comprehensive analysis of their collective influence. A holistic understanding of these factors is crucial for designing targeted interventions to enhance parental well-being and foster greater social inclusion.

To address this gap, the present study adopts the ecological model of social inclusion [3], which conceptualizes social inclusion as a multidimensional and dynamic process influenced by factors at the individual, interpersonal, organizational, community, and socio-political levels. Compared to other theoretical frameworks, the ecological model offers a comprehensive lens to capture these multiple layers, making it especially suitable for complex caregiving contexts [26, 27]. Given the blurred boundaries between levels and undefined specific variables, we classify the associated factors into two broad categories: individual and external factors. This simplified classification is consistent with prior research that has adopted a similar approach to enhance conceptual clarity and operational feasibility [28, 29]. These ecological conditions can either facilitate or impede social inclusion of parents of children with ASD. Therefore, understanding these factors is crucial for developing effective interventions to support social inclusion of parents of children with ASD.

Additionally, previous research has identified differences between fathers and mothers of children with ASD in terms of parenting stress and positive experiences [30, 31]. Beyond ASD, research in the general population suggests that mothers and fathers differ systematically in social participation, access to social resources, and responses to caregiving responsibilities [32, 33], which may shape opportunities for social engagement. These findings indicate that relevant factors of social inclusion may operate differently for mothers and fathers of children with ASD. Therefore, we hypothesize that there are gender-specific differences in factors influencing social inclusion, and identifying them could inform the development of tailored, gender-sensitive interventions to better support both parents.

The objective of this study is to assess social inclusion of parents of children with ASD in China, and to explore the various factors that influence it. Based on the ecological model and prior literature, we hypothesize that both individual factors (self-esteem, hope, and parenting sense of competence) and external factors (intergroup relations, family function, social support, and perceived discrimination) influence social inclusion among parents of children with ASD. Given the intensive caregiving demands and societal challenges faced by this population, we expect that certain external factors – particularly social support, family function, and perceived discrimination – may exert a more pronounced influence than individual factors such as self-esteem or hope. Examining the relative impact of these factors not only addresses gaps in the literature but also provides practical guidance for interventions aimed at enhancing social inclusion in this high-need population.

## Methods

### Study design and participants

A multicenter cross-sectional survey was conducted at 33 rehabilitation centers for children with ASD across 14 prefecture-level divisions in Hunan Province, China, from July 2023 to August 2024. This study was reported following the Strengthening the Reporting of Observational Studies in Epidemiology (STROBE) guidelines [34].

The inclusion criteria were: (1) Being a parent and the primary caregiver of a child diagnosed with ASD according to DSM-5 criteria [1], and (2) having a child with ASD aged 3–14 years [35, 36]. Parents who completed the questionnaire incompletely were excluded from the analysis.

Participant flow. A total of 1,200 parents were invited to participate in the study. Of these, 1050 parents provided informed consent and completed the survey, yielding a response rate of 87.5%. After eligibility screening, 43 parents were excluded because their children were younger than 3 years or older than 14 years (*n* = 32), or due to missing data (*n* = 11). The final analytic sample consisted of 1007 parents.

All participants provided written informed consent prior to participation, and all data were anonymized to ensure confidentiality and privacy. Ethical approval was obtained from the Ethics Committee of Hunan Provincial Brain Hospital (No. 2023-K-001).

### Data collection

A convenience sampling method was employed, recruiting parents from 33 rehabilitation centers for children with ASD. The survey was organized by Guardians of the Stars, a nonprofit project team consisting of one graduate student and 21 undergraduate volunteers from the School of Nursing, Hunan University of Chinese Medicine.

Each participant was individually assessed in a quiet, well-lit room at the rehabilitation center or via an online platform (Sojump, https://www.sojump.com). Parents first provided informed consent and completed demographic questions. Information regarding the diagnosis and severity of ASD was verified by the children’s psychiatrist based on DSM-5 criteria [1, 37]. Subsequently, parents completed a series of standardized questionnaires under the guidance of trained research team members, while another staff member cared for the child to ensure minimal distraction. Each session lasted approximately 30–45 min.

### Measurements of risk factors

#### Sociodemographic characteristics

Parents provided sociodemographic information, including gender, age (years), employment status (employed/unemployed), educational level, marital status (married/others), place of residence, and average monthly family income (RMB, ¥). Data on children were also collected as reported by parents, including gender, age (years), duration of ASD (years), duration of rehabilitation training (months), severity of ASD symptoms, attended a mainstream school or not, whether the child was the only child in the family, and whether siblings had similar developmental disorders.

#### Self-esteem

Self-esteem was assessed using the Self-Esteem Scale (SES) [38], which was translated and validated in Chinese by Ji and Yu [39]. The SES is a unidimensional self-report instrument consisting of 10 items rated on a 4-point Likert scale (1 = not at all, 4 = yes definitely). Example items include “On the whole, I am satisfied with myself” and “I feel I do not have much to be proud of” (reverse scored). Four items (Items 3, 5, 9, and 10) are reverse scored and were recoded prior to analysis. Total scores range from 10 to 40, with higher scores indicating higher self-esteem. The Chinese version of the SES has demonstrated good reliability and validity in previous studies [40], and the Cronbach’ s α coefficient in the present study was 0.85.

#### Hope

Hope was measured using the Herth Hope Index (HHI) [41], translated and adapted into Chinese by Chan et al. [42]. The 12-item scale has three dimensions: positive attitude toward the present and future, taking positive actions, and maintaining close relationships with others. Items are rated on a 4-point Likert scale (1 = strongly disagree, 4 = strongly agree), with higher scores indicating higher hope. Example items include “I have a positive outlook toward life” and “I feel scared about my future” (reverse scored). Two items (Items 3 and 6) are reverse scored and were recoded prior to analysis. Total scores range from 12 to 48. The Chinese version of the HHI has shown good reliability and validity [42], with a Cronbach’s α coefficient of 0.89 in the present study.

#### Parenting sense of competence

Parental self-perceived competence was measured using the Parenting Sense of Competence (PSOC) Scale [43], revised into Chinese by Peng et al. [44]. The 12-item scale includes two dimensions: parenting efficacy and satisfaction. Items are rated on a 4-point Likert scale (1 = strongly disagree, 4 = strongly agree), with higher scores indicating greater perceived competence. Example items include “I meet by own personal expectations for expertise in caring for my child” and “Being a parent makes me tense and anxious” (reverse scored). Reverse-scored items (Items 1, 2,3, 6,7 and 12) were recoded prior to analysis. Total scores range from 12 to 48. In the present study, the Cronbach’s α coefficient for the scale was 0.72.

#### Intergroup relations

Intergroup relations were assessed using a modified Social Distance Scale adapted by Hu et al. [45] from Bogardus et al. [46]. The 5-item scale evaluates willingness to interact with out-group members (primarily parents of typically developing children) on a 5-point Likert scale (1 = very unwilling, 5 = very willing), with lower scores indicating weaker willingness. Example items include “Would you be willing to make friends with parents of typically developing children?”. No items are reverse scored. Total score range: 5–25. The adapted social distance scale has demonstrated good reliability and validity in parents of children with ASD [45], with a Cronbach’s α coefficient of 0.90 in the current study.

#### Family function

Family function was measured using the the Family APGAR Index (APGAR) [47], Chinese version. The 5-item scale covers adaptability, partnership, growth, affection, and resolve. Items are rated 0 (almost never) to 2 (often), with higher scores indicating better family functioning. Example items include “I am satisfied that I can turn to my family for help when something is troubling me”. No items are reverse scored. Total score range: 0–10. In this study, the Cronbach’s α coefficient for the scale was 0.90.

#### Social support

Social support was assessed using the Social Support Scale [48], which is a reliable and valid tool for assessing social support in families of children with ASD. The scale consists of 14 items distributed across three dimensions: emotional support, instrumental support, and informational support. Items are rated 1 (none) to 4 (very much), with higher scores indicating higher perceived social support. Example items include “I have someone I can talk to when I encounter difficulties” and “There is someone who can help take care of my child at home.” No items are reverse scored. Total score range: 14–56. The Chinese version has demonstrated good reliability and construct validity in caregivers of children with ASD [49], with a Cronbach’s α coefficient of 0.94 in the current study.

#### Perceived discrimination

Perceived discrimination was measured using the Perceived Discrimination Scale for Parents of Children with ASD [50]. This 10-item scale consists of two dimensions: discrimination perception and discrimination attribution. Items are rated 1 (not at all) to 4 (yes definitely), higher scores indicate higher perceived discrimination. Example item: “Being a parent of a child with autism (ASD) feels limiting to me.” No items are reverse scored. Total score range: 10–40. Validation studies support reliability in Chinese caregivers of children with ASD [50], with a Cronbach’s α coefficient of 0.90 in the current study.

### Measurements of outcome—social inclusion of parents

Social inclusion was measured using the Social Inclusion Scale (SIS) [51, 52], which evaluates parents’ social contact, social acceptance, participation in activities, and subjective feelings over the past month. The Chinese version of SIS consists of 19 items rated on a 4-point Likert scale (1 = not at all, 4 = yes definitely), with higher scores indicating greater social inclusion. Example items include: “I have felt terribly alone and isolated” (reverse scored) and “I have friends I see or talk to every week”. Reverse-scored items (Items 1, 11, 12, and 17) were recoded prior to analysis. Total scores range from 19 to 76. The Chinese version of the SIS has shown good reliability and validity [52], with a Cronbach’s α coefficient of 0.88 in the present study.

### Statistical analyses

Normality of the data was assessed by Shapiro-Wilk test. For normally distributed continuous variables, results were shown as mean (standard deviation, SD). Non-normally distributed variables were summarized as median (interquartile spacing, IQR). Categorical variables were reported using frequencies and percentages.

Univariate statistical analyses were conducted to assess a preliminary understanding of associations between the independent variable and the dependent variable (Social inclusion). For demographic data, independent samples t-tests or Mann-Whitney U-tests were used for binary categorical variables, one-way ANOVA or Kruskal-Wallis H-tests were used for multi-categorical variables, and Pearson correlation analyses or Spearman rank correlation analyses were used for continuous variables. Due to the normal distribution of the scales’ data, we calculated the Pearson correlation and generated the heatmap.

Based on the social inclusion ecological model and prior evidence, variables that were statistically significant (*P* < 0.05) in the univariate analysis for the total, father, or mother models were included in the multivariate linear regression analysis. We evaluated the clustering effect and found that the between-center variance was estimated as 0, yielding ICC = 0.000, indicating that the clustering effect was not significant [53]. Therefore, we employed multivariate linear regression analysis rather than multilevel regression modelling.

We examined the potential associated factors of social inclusion at the individual level (self-esteem, hope, and parenting sense of competence) and external level (intergroup relations, family function, social support, and perceived discrimination) based on the ecological model of social inclusion [3]. Sociodemographic variables (e.g., parents age, education, employment, family income, residence, children age, and severity of ASD symptoms) were included as covariates in all multivariable models to adjust for confounding. To assess effect modification by gender, we included interaction terms between each predictor and gender in the multivariable regression model and conducted subgroup analyses. For these gender-specific analyses, we included all variables that were significant in any of the three models (overall sample, mother-only sample, or father-only sample) to ensure consistent comparison across groups.

Results were reported with unstandardized regression coefficients (B), and *P* value. The adjusted coefficient of determination (adjusted R^2^) was used to represent the explanatory power of the regression model for the dependent variable. Multicollinearity was assessed by variance inflation factor (VIF) and the variable was removed if it had VIF > 5. Statistical analysis was performed using SPSS (version 28.0) and R 4.2.3. A two-sided significance level of *P* < 0.05 was used.

## Results

### Characteristics of parents and their children with ASD

A total of 1007 parents of children with ASD participated in the survey. Among them, 88.1% were female, with ages ranging from 22 to 54 years. The majority (71.6%) were unemployed, and 17.8% of families had an average monthly family income of ≤ 3,000 RMB (Table 1). Regarding the children, 79.8% were male, with ages ranging from 3 to 14 years. A total of 52.5% had moderate to severe ASD, and 64.5% were not enrolled in mainstream schools.

**Table 1:** Sociodemographic characteristics of the participants and their children with autism spectrum disorder (*n* = 1007)

Variables, *n* (%)		All Parents (*n* = 1007)	Father (*n* = 120)	Mother (*n* = 887)
Characteristics of participants (Parents)				
Age (years)	< 40	785 (78.0)	75 (62.5)	710 (80.0)
	≥ 40	222 (22.0)	45 (37.5)	177 (20.0)
Employment status	Employed	286 (28.4)	70 (58.3)	216 (24.4)
	Unemployed	721 (71.6)	50 (41.7)	671 (75.6)
Education level	Middle school and below	190 (18.9)	23 (19.2)	167 (18.8)
	High school/Junior college	394 (39.1)	39 (32.5)	355 (40.0)
	Undergraduate/College and above	423 (42.0)	58 (48.3)	365 (41.1)
Marital status	Married	971 (96.4)	109 (90.8)	862 (97.2)
	Others	36 (3.6)	11 (9.2)	25 (2.8)
Place of residence	Rural	392 (38.9)	59 (49.2)	333 (37.5)
	Urban	615 (61.1)	61 (50.8)	554 (62.5)
Average monthly family income (RMB, ¥)	≤ 3,000	179 (17.8)	28 (23.3)	151 (17.0)
	3,001–6,000	440 (43.7)	47 (39.2)	393 (44.3)
	6,001–9,000	201 (20.0)	22 (18.3)	179 (20.2)
	≥ 9,001	187 (18.6)	23 (19.2)	164 (18.5)
Characteristics of children with autism spectrum disorders				
Gender	Male	804 (79.8)	100 (83.3)	704 (79.4)
	Female	203 (20.2)	20 (16.7)	183 (20.6)
Age (years)	< 6	475 (47.2)	46 (38.3)	429 (48.4)
	≥ 6	532 (52.9)	74 (61.7)	458 (51.6)
Duration of ASD (years)	≤ 3	559 (55.5)	54 (45.0)	505 (56.9)
	> 3	448 (44.5)	66 (55.0)	382 (43.1)
Duration of rehabilitation training (months)	≤ 24	525 (52.1)	56 (46.7)	469 (52.9)
	> 24	482 (47.9)	64 (53.3)	418 (47.1)
Severity of ASD symptoms	Mild	478 (47.5)	56 (46.7)	422 (47.6)
	Moderate and Severe	529 (52.5)	64 (53.3)	465 (52.4)
Children were enrolled in a mainstream school	Yes	357 (35.5)	38 (31.7)	319 (36.0)
	No	650 (64.5)	82 (68.3)	568 (64.0)
The child was the only child in the family	Yes	362 (35.9)	54 (45.0)	308 (34.7)
	No	645 (64.1)	66 (55.0)	579 (65.3)
Siblings with similar developmental disorders	Yes	30 (3.0)	3 (2.5)	27 (3.0)
	No	615 (61.1)	63 (52.5)	552 (62.2)
	Without siblings	362 (35.9)	54 (45.0)	308 (34.7)

### Social inclusion and associated factors scores of parents of children with ASD

Table 2 shows the descriptive statistics for the scale-based independent variables of parents of children with ASD. The mean social inclusion score (SD) of all parents was 54.16 (8.15), the social inclusion score of fathers and mothers were 55.98 (8.42) and 53.91 (8.08), respectively. Participants reported a mean self-esteem score of 29.21 (4.43), a mean hope score of 36.38 (5.09), and a mean parenting sense of competence score of 30.83 (4.47). The mean scores for intergroup relations and family functioning were 21.30 (3.04) and 6.21 (2.63), respectively. Additionally, the mean social support score was 31.82 (8.74), while the mean perceived discrimination score was 19.17 (5.88).

**Table 2:** Risk factors measurements scores of parents of children with autism spectrum disorder (*n* = 1007)

Measurements^a^	Scores, Mean (SD)		
	All Parents (*n* = 1007)	Father (*n* = 120)	Mother (*n* = 887)
Self-esteem	29.21 (4.43)	30.03 (4.60)	29.10 (4.40)
Hope	36.38 (5.09)	37.31 (5.38)	36.26 (5.04)
Parenting sense of competence	30.83 (4.47)	32.28 (5.13)	30.63 (4.34)
Intergroup relations	21.30 (3.04)	21.03 (3.04)	21.33 (3.04)
Family function	6.21 (2.63)	6.73 (2.62)	6.14 (2.63)
Social support	31.82 (8.74)	32.34 (8.68)	31.75 (8.75)
Perceived discrimination	19.17 (5.88)	17.96 (5.55)	19.33 (5.91)

^a^Measurement Score interpretation: Self-esteem (10–40, higher scores indicate higher self-esteem); Hope (4–48, higher scores indicate greater hope); Parenting sense of competence (4–48, higher scores indicate higher parenting sense of competence); Intergroup relations (5–25, higher scores indicate better intergroup relationships); Family function (0–10, higher scores indicate better family functioning); Social support (4–56, higher scores indicate greater perceived social support); Perceived discrimination (4–40, higher scores indicate higher levels of perceived discrimination)

### The univariate analyses of the potential factors associated with social inclusion of parents of children with ASD

In the univariate analysis, higher social inclusion scores in parents were significantly associated with children’s age younger than 6 years (*P* = 0.048), lower severity of illness in children with ASD (*P* < 0.001), children being enrolled in mainstream schools (*P* < 0.001), male parents (*P* = 0.009), parents being employed (*P* < 0.001), higher parental education level (*P* < 0.001), urban residence (*P* < 0.001), and higher family average monthly income (*P* < 0.001) (Table 3). The results of the univariate analysis of mothers were consistent with those of all parents, except that age less than 40 years was also associated with high social inclusion scores (*P* = 0.03). However, only high education level (*P* = 0.004), urban residence (*P* = 0.01), and low severity ASD symptoms in children (*P* = 0.01) significantly influenced high social inclusion of fathers.

**Fig. 1:**
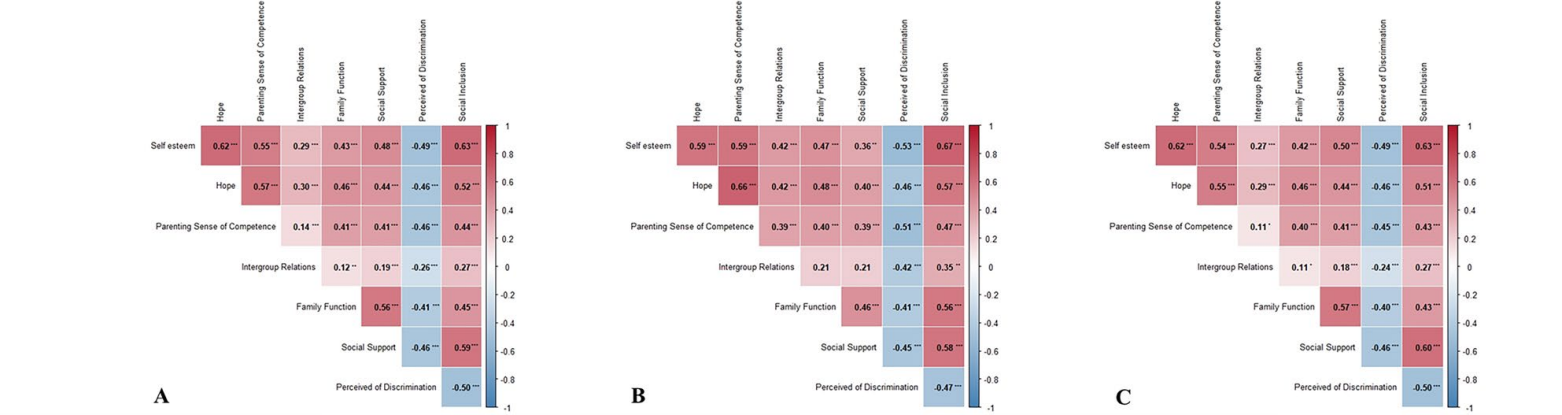
Heatmap of parents’ social inclusion and scale-based independent variables Cell colors indicate both the direction and strength of correlations, with red representing positive correlations and blue representing negative correlations, where deeper colors signify stronger relationships. **A** Overall sample, **B** father-only sample, **C** mother-only sample. * *P* < 0.05; ** *P* < 0.01; *** *P* < 0.001

Pearson correlation analyses were conducted between social inclusion and scale-based independent variables, as shown in Fig. 1. In the overall sample, mother-only sample, and father-only sample, self-esteem, hope, parenting sense of competence, intergroup relations, family function, and social support were positively correlated with social inclusion, and perceived discrimination was inversely correlated with social inclusion.

**Table 3:** Univariate analysis ofsociodemographic characteristics and independent variables related to parents’social inclusion

Variables		All Parents (*n* = 1007)		Father (*n* = 120)		Mother (*n* = 887)	
		Social inclusion, Mean (SD)	*P* value	Social inclusion, Mean (SD)	*P* value	Social inclusion, Mean (SD)	*P* value
Characteristics of participants (Parents)							
Gender	Male	55.98 (8.48)	0.009	-	-	-	-
	Female	53.91 (8.08)		-	-	-	-
Age (years)	< 40	54.36 (8.03)	0.15	55.84 (8.61)	0.81	54.20 (7.96)	0.03
	≥ 40	53.45 (8.56)		56.22 (8.35)		52.75 (8.49)	
Employment status	Employed	57.11 (8.63)	< 0.001	56.53 (8.22)	0.41	57.30 (8.77)	< 0.001
	Unemployed	52.99 (7.65)		55.22 (8.87)		52.82 (7.53)	
Educational level	Middle school and below	50.73 (7.57)	< 0.001	51.87 (8.37)	0.004	50.57 (7.47)	< 0.001
	High school/Junior college	53.30 (7.18)		54.82 (7.65)		53.14 (7.12)	
	Undergraduate/College and above	56.50 (8.56)		58.40 (8.40)		56.19 (8.56)	
Marital status	Married	54.18 (8.08)	0.71	55.88 (8.26)	0.69	53.96 (8.04)	0.28
	Others	53.67 (10.02)		57.00 (10.88)		52.20 (9.47)	
Place of residence	Rural	52.46 (8.03)	< 0.001	54.03 (9.32)	0.01	54.95 (8.10)	< 0.001
	Urban	55.24 (8.05)		57.87 (7.17)		52.18 (7.76)	
Average monthly family income	≤ 3,000	51.59 (9.23)	< 0.001	53.29 (9.90)	0.09	51.28 (9.10)	< 0.001
	3,001–6,000	53.68 (7.81)		55.83 (9.04)		53.42 (7.63)	
	6,001–9,000	54.80 (7.08)		56.27 (5.29)		54.62 (7.26)	
	≥ 9,001	57.04 (8.02)		59.30 (7.11)		56.73 (8.11)	
Characteristics of children with autism spectrum disorders							
Gender	Male	54.11 (8.18)	0.72	56.44 (8.31)	0.19	53.78 (8.12)	0.34
	Female	54.34 (8.05)		53.70 (9.20)		54.42 (7.93)	
Age	< 6	54.69 (8.09)	0.048	57.87 (9.04)	0.05	54.35 (7.92)	0.11
	≥ 6	53.68 (8.19)		54.81 (7.96)		53.50 (8.22)	
Duration of ASD (year)	≤ 3	54.32 (7.80)	0.48	56.54 (8.61)	0.52	54.08 (7.68)	0.47
	> 3	53.96 (8.58)		55.53 (8.42)		53.68 (8.59)	
Duration of rehabilitation training (month)	≤ 24	54.20 (7.96)	0.86	56.32 (8.56)	0.69	53.95 (7.86)	0.88
	> 24	54.11 (8.37)		55.69 (8.47)		53.87 (8.33)	
Severity of ASD symptoms	Mild	55.51 (7.84)	< 0.001	58.04 (8.19)	0.01	55.18 (7.74)	< 0.001
	Moderate and Severe	52.93 (8.25)		54.19 (8.39)		52.76 (8.22)	
Children were enrolled in a mainstream school	Yes	55.56 (7.84)	< 0.001	56.13 (7.93)	0.90	55.49 (7.83)	< 0.001
	No	53.39 (8.23)		55.91 (8.78)		53.02 (8.09)	
The child was the only child in the family	Yes	53.84 (7.91)	0.10	56.02 (8.75)	0.97	54.50 (8.52)	0.11
	No	54.73 (8.56)		55.95 (8.33)		53.60 (7.83)	
Siblings with similar developmental disorders	Yes	51.37 (9.53)	0.08	61.33 (11.24)	0.26	50.26 (8.87)	0.02
	No	53.96 (7.81)		55.70 (8.19)		53.76 (7.75)	

### The multivariate regression analysis of the potential factors associated with social inclusion of parents of children with ASD

Prior to conducting the multivariate regression analysis, all key assumptions of the model were met. The VIF values for each variable in all linear regression models were consistently below 5, ranging between 1.05 and 4.17, indicating that no adjustments were necessary for the variables. Residual diagnostics (histogram and normal P–P plots of standardized residuals, and a scatterplot) suggested that residuals were approximately normally distributed and that no clear heteroscedasticity was present (Supplementary file 1). The Durbin-Watson statistics for the overall, father and mother models were 1.93, 1.94, and 1.91 respectively, suggesting independence of observations.

The multivariate regression analysis revealed several factors significantly associated with social inclusion of parents of children with ASD (VIF: 1.05 to 2.52, Table 4). Among individual level factors, higher education level (Undergraduate/College and above) (B = 1.764, *P* = 0.02), self-esteem (B = 0.472, *P* < 0.001), and hope (B = 0.139, *P* = 0.03) demonstrated positive impact on social inclusion.

At the external level, intergroup relations (B = 0.271, *P* = 0.001), and social support (B = 0.238, *P* < 0.001) demonstrated significant positive associations with social inclusion. Conversely, perceived discrimination (B=-0.189, *P* < 0.001) showed a significant negative association with social inclusion. Overall, the model has an explanatory power of 49.5% for social inclusion (Table 4).

Interaction terms between gender and each key predictor were not statistically significant (all *P* for interaction > 0.05), indicating no evidence of effect modification by gender in the regression model. We nevertheless conducted exploratory gender-stratified analyses to describe potential patterns (Supplementary file 1). Subgroup analysis revealed that the higher self-esteem (B = 0.815, *P* = 0.01) and better social support (B = 0.351, *P* = 0.01), the better social inclusion of fathers. The regression equation for fathers demonstrated the moderate explanatory power (Adjusted R^2^ = 0.492, VIF: 1.29 to 4.17). For mothers, results were generally consistent with those observed in the overall parent group, except for the individual level of hope (B = 0.095, *P* = 0.16), which had no significant impact on social inclusion. These variables explain 48.7% of mothers’ social inclusion (VIF: 1.09 to 2.54, Table 4). To assess estimate stability, we performed bootstrap internal validation for the overall, father-only, and mother-only regression models. The key predictors retained consistent directions and remained statistically significant, indicating robust and stable associations.

**Table 4:** Associations between social inclusion and associated factors of parents of children with autism spectrum disorder

Subgroup	All Parents (*n* = 1007)			Father (*n* = 120)			Mother (*n* = 887)		
Factors^a^	Unstandardized coefficients B	95% CI for B	*P*	Unstandardized coefficients B	95% CI for B	*P*	Unstandardized coefficients B	95% CI for B	*P*
Individual level									
Gender of parents									
Male	Ref			-	-	-	-	-	-
Female	-1.329	-2.829 to 0.171	0.08	-	-	-	-	-	-
Age of parents (years)									
< 40	Ref			Ref			Ref		
≥ 40	-0.167	-1.203 to 0.869	0.75	-1.098	-5.272 to 3.076	0.60	-0.049	-1.165 to 1.066	0.93
Employment status									
Unemployed	Ref			Ref			Ref		
Employed	-0.092	-1.220 to 1.036	0.87	-0.261	-3.771 to 3.249	0.88	-0.048	-1.279 to 1.182	0.94
Educational level									
Middle school and below	Ref			Ref			Ref		
High school/Junior college	0.769	-0.439 to 1.976	0.21	3.809	-1.637 to 9.255	0.17	0.657	-0.610 to 1.923	0.31
Undergraduate/College and above	1.764	0.325 to 3.204	0.02	4.391	-1.657 to 10.439	0.15	1.582	0.048 to 3.115	0.04
Place of residence									
Urban	Ref			Ref			Ref		
Rural	-0.580	-1.629 to 0.469	0.28	-0.286	-4.453 to 3.881	0.89	-0.569	-1.674 to 0.536	0.31
Average monthly family income									
≤ 3000	Ref			Ref			Ref		
3001–6000	0.092	-1.146 to 1.331	0.88	1.055	-5.070 to 7.179	0.73	-0.164	-1.463 to 1.136	0.81
6001–9000	-0.237	-1.754 to 1.280	0.76	2.875	-3.172 to 8.921	0.34	-0.695	-2.314 to 0.923	0.40
≥ 9001	0.676	-0.978 to 2.330	0.42	-0.347	-6.920 to 6.227	0.92	0.681	-1.075 to 2.437	0.45
Age of children (years)									
< 6	Ref			Ref			Ref		
≥ 6	-0.375	-1.283 to 0.533	0.42	1.545	-1.940 to 5.030	0.38	-0.586	-1.544 to 0.373	0.23
Severity of children’s ASD symptoms									
Mild	Ref			Ref			Ref		
Moderate and Severe	0.082	-0.869 to 1.034	0.87	-1.469	-4.871 to 1.933	0.39	0.277	-0.730 to 1.284	0.59
Children were enrolled in a mainstream school									
No	Ref			Ref			Ref		
Yes	0.544	-0.467 to 1.555	0.29	-1.093	-4.906 to 2.721	0.57	0.801	-0.268 to 1.870	0.14
Siblings with similar developmental disorders									
No	Ref			Ref			Ref		
Yes	-0.036	-2.148 to 2.075	0.97	1.970	-6.407 to 10.347	0.64	-0.150	-2.373 to 2.073	0.90
Self-esteem	0.472	0.326 to 0.618	< 0.001	0.815	0.197 to 1.433	0.01	0.468	0.315 to 0.620	< 0.001
Hope	0.139	0.014 to 0.265	0.03	0.396	-0.103 to 0.895	0.12	0.095	-0.038 to 0.228	0.16
Parenting sense of competence	0.128	-0.005 to 0.262	0.06	-0.098	-0.666 to 0.470	0.73	0.140	-0.001 to 0.281	0.05
External level									
Intergroup relations	0.271	0.106 to 0.437	0.001	0.135	-0.516 to 0.786	0.68	0.295	0.118 to 0.472	0.001
Family function	0.056	-0.160 to 0.271	0.61	-0.062	-0.961 to 0.836	0.89	0.052	-0.172 to 0.277	0.65
Social support	0.238	0.168 to 0.308	< 0.001	0.351	0.084 to 0.618	0.01	0.234	0.160 to 0.308	< 0.001
Perceived discrimination	-0.189	-0.290 to -0.089	< 0.001	-0.066	-0.491 to 0.359	0.76	-0.211	-0.316 to -0.107	< 0.001

CI: confidence intervals

^a^All analyses were conducted using multivariate linear regression models, controlling for sociodemographic variables

## Discussion

In this multicenter cross-sectional study, we identified both individual and external factors associated with social inclusion among 1007 parents of children with ASD. Overall, our findings partially supported our prior expectation that external factors would play a particularly significant role. Social support and intergroup relations were associated with social inclusion. At the same time, individual psychological resources also mattered. Self-esteem and hope were positively related to social inclusion. Although formal interaction tests did not reveal gender effects on social inclusion, exploratory stratified analyses indicated different patterns of association. Fathers’ social inclusion was most strongly linked to self-esteem and social support, while mothers’ social inclusion was more closely associated with intergroup relations, perceived discrimination, and educational level.

Among the participants in this study, 88.1% of the caregivers were mothers, which is consistent with prior research [54]. Subsequent discussion focuses on the factors influencing social inclusion for both mothers and fathers. We found that higher education level, especially undergraduate or college and above education, significantly increases the social inclusion of parents. This was also found in the subgroup analysis of the effects of social inclusion of mothers. These findings were consistent with previous research that demonstrated higher levels of education were associated with better social inclusion of parents of children with ASD [55]. Higher education can provide parents with greater knowledge and resources, enable parents to have stronger communication skills and self-advocacy skills [56]. These skills are particularly important in overcoming the stigma and misunderstanding that parents of children with ASD often face in social environments. Mothers of children with ASD often face intense scrutiny and judgment of their parenting abilities, which can lead to increased social isolation [57]. Higher education can provide mothers with greater confidence and self-efficacy to address these social challenges and counteract internalized stigma [58]. These findings underscore the importance of designing customized interventions that consider parents’ educational levels and caregiver gender in future efforts to promote the social inclusion of parents of children with ASD.

Parents of children with ASD often face long-term or even lifelong caregiving responsibilities due to the unknown causes of the disorder and the lack of a clear treatment [59]. In China, the majority of existing ASD assistance policies are limited to children aged 0–14 years, leading to widespread parental concerns and uncertainties about their children’s future [60]. In this context, self-esteem and hope play key roles in enhancing parents’ social inclusion, as they are important psychological resources that support parents’ ability to engage in social interactions and build support networks. Self-esteem and hope were positively associated with parents’ social inclusion, consistent with previous research that self-esteem and hope are psychological resources that enhance an individual’s ability to engage in social interactions and build support networks [61, 62]. It is plausible that self-esteem and hope operate as activation resources that lower the threshold for social approach behaviors, helping parents shift from more passive reliance on close family to proactively engaging with broader networks and community resources, thereby translating psychological resilience into observable social inclusion [63–66]. Research shows that hope reduces stress, enhances coping strategies and develops a sense of control over the future, despite uncertainty. Beyond reflecting positive affect, hope may be particularly salient because it links future-oriented meaning with pathways and agency. Parents with higher hope may be more likely to initiate problem-solving steps, such as seeking information, negotiating services, or joining peer groups, which can accumulate into sustained social connectedness over time [62, 67]. These findings emphasize the importance of personal psychological resources in promoting social inclusion in parents of children with ASD. Interventions aimed at increasing self-esteem and hope, particularly focusing on positive self-evaluation, future orientation, and relationship maintenance skills, may be particularly beneficial in improving social inclusion for parents.

Our study showed the positive impact of intergroup relations and social support on parents’ social inclusion. These findings suggest that social inclusion may be shaped not only by parents’ internal resources, but also by the social permeability of the environments they navigate, such as whether everyday interactions with out-group members are welcoming and whether reliable support can be mobilized when needed [68]. One plausible mechanism is that positive contact with parents of typically developing children functions as a bridge to mainstream community life. It can normalize parents’ caregiving experiences, reduce anticipated stigma, and expand access to practical information and opportunities that may otherwise remain within ASD-specific circles [69]. This is important because parents may have strong bonds within ASD peer networks yet still feel excluded from broader community participation, intergroup contact may be a key pathway from bonding support to bridging inclusion. Social support makes participation feasible by sharing the caregiving load, enabling time and mobility, and providing reassurance that setbacks will be met with help rather than judgment [70], providing emotional and practical support that is often lacking in formal services, and can relieve the stress that parents experience when caring for a child with ASD, increase their life satisfaction, thereby improving their social inclusion [71]. Practically, these results point to the potential value of community-based programs that create structured, positive intergroup contact alongside support mechanisms that reduce participation barriers, rather than relying solely on parent-focused psychological interventions.

Our study found that perceived discrimination had a negative effect on parents’ social inclusion. This pattern is consistent with stress–coping perspectives in which stigma-related experiences can function as a chronic stressor that shapes appraisal, coping, and subsequent social behavior [72]. In Asian cultures, including China, collateral stigma and perceived discrimination may be particularly pronounced due to cultural beliefs, as well as a lack of awareness about ASD among relatives and the broader society [73]. When parents perceive discrimination, they may internalize the stigma, integrating it into their self-perception and value system. This internalization can intensify their perceived stress and negative emotions, further exacerbating feelings of isolation and self-doubt [74]. To prevent negative reactions, parents adopt social withdrawal behaviors as a protective measure, reinforcing social anxiety and limiting their opportunities for social inclusion. Given these challenges, healthcare providers and society need to become more aware of the stigma and challenges these parents face, provide them with more compassionate healthcare, and create better environments for families and children with ASD. Enhancing social inclusion may require measures at both the individual and community levels. Alongside providing psychosocial support to parents, implementing anti-stigma initiatives, raising awareness about ASD, and introducing accessibility measures in schools and communities, can help dismantle barriers created by stigma. These efforts can create a safer social environment for families affected by ASD.

Although the interaction term between gender and the main factors was not statistically significant, subgroup analyses revealed significant differences between fathers and mothers in the factors affecting social inclusion. Intergroup relations and perceived discrimination had a significant effect on mothers’ social inclusion when compared to fathers. In addition to the relatively small sample size of fathers, this could be because of the different roles that fathers and mothers assume when caring for children with ASD. Mothers are more likely to be the primary caregivers for their children, and caregiver responsibilities have increased [75]. Mothers are most often in the public eye when their children misbehave at school and in the community, intensifying the feelings of shame they experience [76]. Mothers must address their children’s behavioral challenges as well as the social judgments of others who may not be aware of ASD. These frequent and often stressful public interactions may make mothers sensitive to perceived discrimination, making the quality of intergroup relationships particularly important to their sense of social inclusion [77]. Therefore, interventions tailored to mothers should focus on creating supportive community spaces, providing positive inter-group contact and reducing the overburdening of discrimination experienced by mothers, thereby improving their social inclusion and overall well-being. Although gender-stratified models suggested some differences in which predictors reached statistical significance, formal interaction tests did not support effect modification by gender. Therefore, the significant differences observed between father-only and mother-only models are exploratory and may be due to sampling variability, especially considering that the father sample size is much smaller than the mother sample size. Future studies with balanced samples are needed to more definitively test whether associations differ by gender.

### Practice implications

Promoting the social inclusion of parents of children with ASD is essential as it directly impacts their mental health outcomes, reduces the burden and stress on parents, and improves their ability to effectively support their children [78]. Nurses play a key role in improving the social inclusion of parents of children with ASD because they are uniquely positioned at the intersection of the health care system and community resources to maintain ongoing relationships with families throughout the continuum of care [79]. Theoretically, this study advances the ecological model of social inclusion by highlighting the differentiated effects of individual, interpersonal and structural factors. At the individual psychological level, nurses can incorporate self-esteem and hope screenings into routine home assessments and provide parents with attentive care and social support to help them develop accurate self-perceptions, recognize their internal value, and develop psychological resilience [80]. These interventions can be parent-centered and combined with individualized psychosocial support services that acknowledge the unique challenges faced by parents, effectively address their psychological needs. At the community and societal level, nurses can facilitate parent support groups that not only provide emotional affirmation but also create opportunities for meaningful contact between groups outside of the autism community. By collaborating with community organizations, nurses can help parents engage in positive interactions, improve public perceptions of autism, and reduce stigma and shame, thereby increasing parents’ social inclusion [81]. Healthcare systems could develop policies that provide a complete, long-term system of services for ASD children and parents [82]. Additionally, flexible scheduling and workplace accommodations by society for parents of children with special needs can help address the burden of care that limits social participation.

### Strengths and limitations

There are some strengths of this study that contribute to the understanding of the social inclusion of parents of children with ASD. First, we combined the ecological model with a comprehensive approach to examine individual factors and external factors, which provided a multidimensional understanding of social inclusion. Second, this study utilized a multicenter design to obtain data from different geographic regions and socioeconomic contexts, which enhanced the representativeness and generalizability of the findings. Third, gender-specific subgroup analyses revealed important differences between mothers’ and fathers’ social inclusion, addressing a research gap in the literature.

This study has several limitations. First, participants were recruited using convenience sampling from rehabilitation centers for children with ASD in Hunan Province, China, which may introduce selection bias and limit the generalizability of the findings to families not engaged in rehabilitation services, other regions of China, or different care settings. In addition, potential nonresponse bias cannot be excluded, as caregivers experiencing greater burden or lower service engagement may have been less likely to participate. Second, only parents who were primary caregivers of children diagnosed with ASD were included. Mothers comprised nearly 90% of the sample, resulting in an uneven gender distribution and limiting the generalizability of findings to fathers or to parental dyads. This imbalance may also have reduced statistical power for analyses specific to fathers and precluded examination of family-level caregiving dynamics. Third, all psychosocial variables were self-reported, which may be subject to social desirability bias. Although the instruments demonstrated high internal consistency, the lack of formal psychometric validation within this specific population remains a limitation. Fourth, we did not implement formal false discovery rate control or a pre-specified hierarchical modelling strategy. Although variables were conceptually organized and interpreted according to the ecological framework, future studies should predefine analytic hierarchies and apply internal validation approaches to reduce the risk of overfitting and multiplicity-driven findings. Moreover, despite the multi-center design, cluster-robust or multilevel sensitivity analyses were not conducted and should be considered in future research to assess potential center-level effects. Finally, due to the cross-sectional design, causal relationships between the identified factors and social inclusion cannot be established. Longitudinal studies are warranted to explore potential bidirectional associations. In addition, although this study adopted an ecological model of social inclusion, not all relevant factors could be comprehensively captured, and the findings should be viewed as a foundation for future research.

## Conclusions

This study demonstrates that social inclusion of parents of children with ASD is influenced by multiple factors at the individual and external levels. Higher education level, self-esteem, hope, intergroup relations, and social support are protective factors for social inclusion, while perceived discrimination is the main barrier. In the future, there is a need to develop targeted interventions that address both psychological resources and social barriers, with particular attention to gender-specific needs and experiences.

## Supplementary Information

Below is the link to the electronic supplementary material.

Supplementary Material 1.

## Data Availability

The datasets generated and analysed during the current study are not publicly available due to privacy protection and ethical considerations.

## Abbreviations

ASD: Autism spectrum disorder
SES: The Self-Esteem Scale
HHI: The Herth Hope Index
PSOC: The Parenting Sense of Competence
SIS: The Social Inclusion Scale
SD: Standard deviation
IQR: Interquartile spacing
CI: Confidence intervals
