# Supplementary material for: Factors associated with social inclusion of parents of children with autism spectrum disorder: a multicenter cross-sectional study in China
Source: Child Adolesc Psychiatry Ment Health. 2026 Mar 14;20:61. doi: 10.1186/s13034-026-01065-w (PMC13101307; doi:10.1186/s13034-026-01065-w)
Supplement: Supplementary file 1 — Supplementary Material 1. [file 13034_2026_1065_MOESM1_ESM.docx]

**Supplemental Table** Results of main and interaction effects between gender and key factors on social inclusion of parents of children with autism spectrum disorder

|  | **All Parents (n=1007)** | | **Father (n=120)** | **Mother (n=887)** |
| --- | --- | --- | --- | --- |
|  | **Model 1 (Factors)** | **Model 2^*^ (Gender × Factors)** | **Model 3 (Factors)** | **Model 4 (Factors)** |
| **Internal Factors** | *P* | *P* | *P* | *P* |
| Self-esteem | <0.001 | 0.26 | 0.01 | <0.001 |
| Hope | 0.03 | 0.053 | 0.12 | 0.16 |
| Parenting sense of competence | 0.06 | 0.61 | 0.73 | 0.05 |
| **External level** |  |  |  |  |
| Intergroup relations | 0.001 | 0.82 | 0.68 | 0.001 |
| Family function | 0.61 | 0.22 | 0.89 | 0.65 |
| Social support | <0.001 | 0.35 | 0.01 | <0.001 |
| Perceived discrimination | <0.001 | 0.81 | 0.76 | <0.001 |

^*^ Model 2 includes interaction terms between gender and each factor.

**Supplemental Figure** Results of the normality test for social inclusion


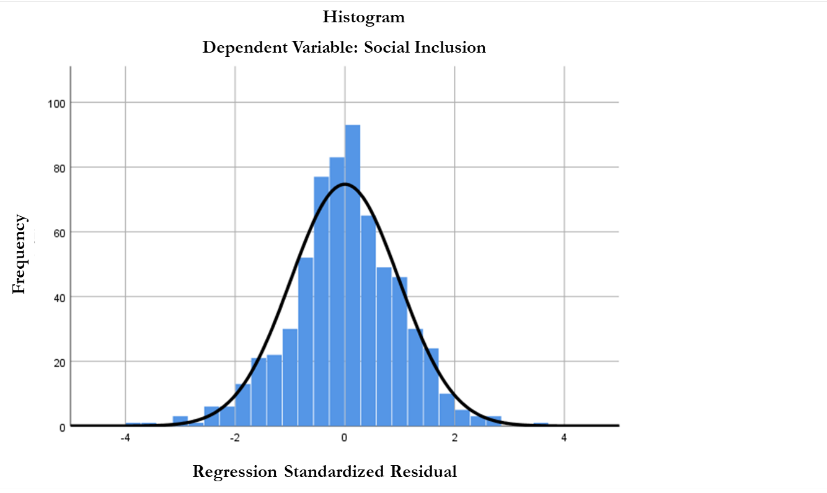


S. Figure 1 Histogram plot of residual


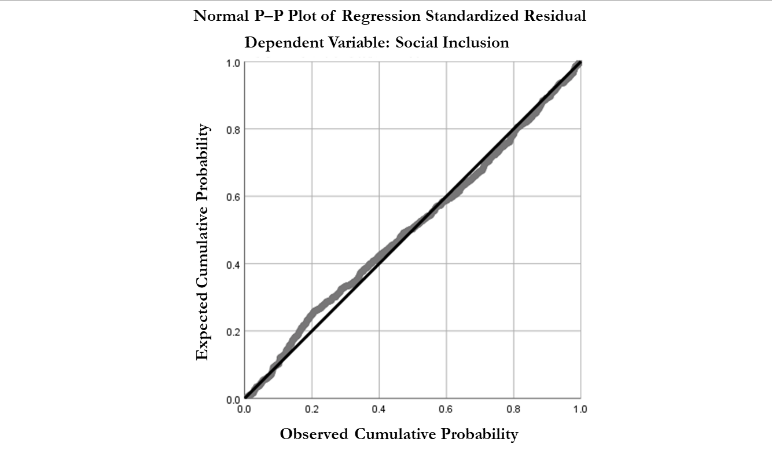


S. Figure 2 Normal P-P plot of regression standardized residual


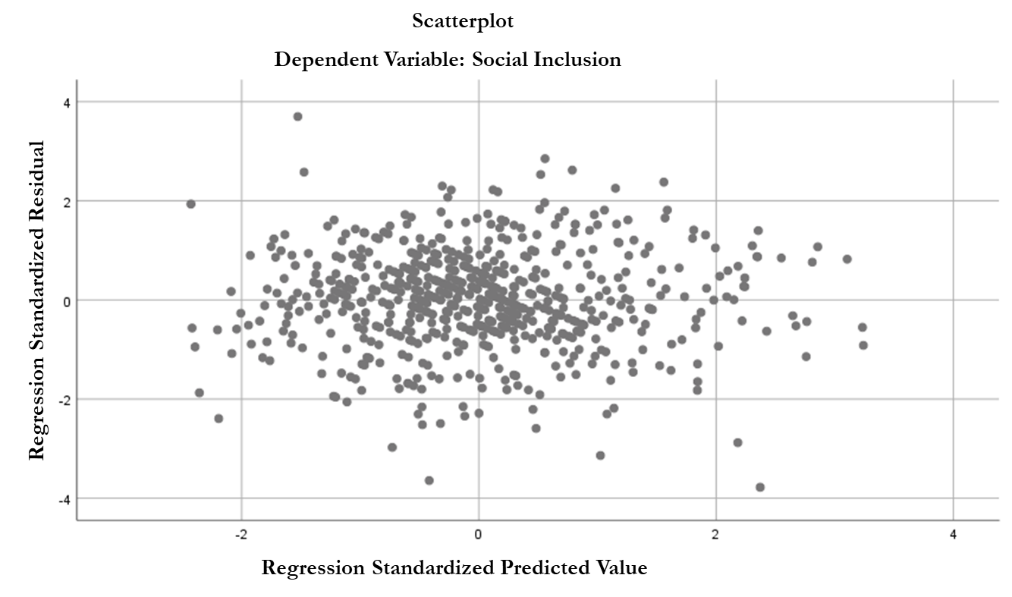


S. Figure 3 Scatterplot of regression standardized residual
